# Genetic polymorphisms of FAS and EVER genes in a Greek population and their susceptibility to cervical cancer: a case control study

**DOI:** 10.1186/s12885-016-2960-3

**Published:** 2016-11-29

**Authors:** Evangelia Pavlidou, Alexandros Daponte, Raquel Egea, Efthimios Dardiotis, Georgios M. Hadjigeorgiou, Antonio Barbadilla, Theodoros Agorastos

**Affiliations:** 1 Department of Gynaecology and Obstetrics, Geneva University Hospitals , Geneva, Switzerland; 2 4th University Clinic of Obstetrics and Gynecology, Aristotle University of Thessaloniki, “Hippokrateion” General Hospital of Thessaloniki, Thessaloniki, Greece; 3 Department of Obstetrics and Gynaecology, Faculty of Medicine, University of Thessaly, University Hospital of Larissa, Larissa, Greece; 4 Institut de Biotecnologia i Biomedicina/Departament de Genètica i de Microbiologia, Universitat Autònoma Barcelona, 08192 Cerdanyola, Barcelona, Spain; 5 Department of Neurology, Laboratory of Neurogenetics, Faculty of Medicine, University of Thessaly, University Hospital of Larissa, Larissa, Greece

**Keywords:** EVER, FAS, Polymorphism, Cervical cancer

## Abstract

**Background:**

The aim of the study was to evaluate the association of two SNPs of EVER1/2 genes’ region (rs2290907, rs16970849) and the FAS-670 polymorphism with the susceptibility to precancerous lesions and cervical cancer in a Greek population.

**Methods:**

Among the 515 women who were included in the statistical analysis, 113 belong to the case group and present with precancerous lesions or cervical cancer (27 with persistent CIN1, 66 with CIN2/3 and 20 with cervical cancer) and 402 belong to the control group. The chi-squared test was used to compare the case and the control groups with an allelic and a genotype-based analysis.

**Results:**

The results of the statistical analysis comparing the case and the control groups for all the SNPs tested were not statistically significant. Borderline significant difference (*p* value = 0.079) was only found by the allelic model between the control group and the CIN1/CIN2 patients’ subgroup for the polymorphism rs16970849. The comparison of the other case subgroups with the control group did not show any statistically significant difference.

**Conclusions:**

None of the SNPs included in the study can be associated with statistical significance with the development of precancerous lesions or cervical cancer.

## Background

Cervical cancer is the third most commonly diagnosed cancer and the fourth leading cause of cancer deaths in women worldwide, accounting for 9% (529,800) of the total new cancer cases and 8% (275,100) of the total cancer deaths among females in 2008 [1]. Despite the screening opportunities offered in the last decades, cervical cancer still remains the second most common type after breast cancer in the European Union in women aged 15–44 years old [2]. The causal relationship between human papillomavirus (HPV) infection and the development of cervical cancer, originally proposed by Prof Zur Hausen [3, 4], has been identified from several studies as one of the highest risk ratios reported for a specific cause of any major human cancer [5, 6]. However, the fact that only a small proportion of the women infected with an oncogene HPV type develop cervical cancer, indicates that other factors may play an important role in the susceptibility for cervical cancer, such as the genetic predisposition [7]. There are several genetic variants associated with the persistence of the HPV infection and the evolution to cervical cancer, mainly by interfering with the host’s immune response [8–10]. The EVER 1 and EVER2 genes (also referred to as TMC6 and TMC8 genes), located on the chromosome 17q25 are two of the genetic polymorphisms which were recently related to cervical cancer. These genes are most widely known for the development of the rare autosomal recessive disease of Epidermodysplasia Verruciformis (EV) which is associated with extremely high sensitivity to HPV infections by HPV types belonging to the beta-genus [11]. Although the exact mechanism of this uncommon susceptibility to HPV infections remains unknown, it has been shown that loss-of-function mutations in TMC6 or TMC8 genes play an important role in most of the EV cases [12]. The TMC6 and TMC8 proteins seem to participate also in the regulation of cellular zinc homeostasis in keratinocytes and lymphocytes, thus they can influence several signal transduction pathways in different cell types [12, 13]. Concerning the association of EVER1/2 genes with the development of cervical cancer, Wang et al. studied 7140 single nucleotide polymorphisms (SNPs) from 305 genes potentially related to the DNA repair mechanisms, the HPV infection and the entry of the HPV virus in the host cells [14]. The study included 416 women with cervical intraepithelial neoplasia grade 3 (CIN3) or cervical cancer, 356 women with persistent HPV infection and 425 controls. The sample consisted of women who participated in the population-based cohort study of women in Guanacaste, Costa Rica [15] and 331 supplemental CIN3 and cancer cases from Guanacaste who were not participants in the natural history study. The SNP rs9893818, which is located near the EVER1/2 genes, was classified among the regions that present a statistically significant association with the progression of the HPV infection to CIN3/cervical cancer [14]. Recently, Castro et al. analysed 22 SNPs across an extended genomic region that harbours TMC6 and TMC8 genes in 2989 patients with CIN3 (93%) or invasive cervical carcinoma (ICC) (7%) and 2281 controls from the Swedish population [16]. The study included two Swedish cohorts. Cohort 1 was based on a nested study within the Northern Sweden Health and Disease Study Cohort [9]. Cohort 2 included women from families with at least two affected women from all of Sweden [17]. Two of the SNPs studied (rs2290907 and rs16970849) were found to be significantly correlated with cervical cancer (CIN3 and ICC) [16].

Another genetic locus widely studied in cervical carcinogenesis is the encoding gene of the cell surface death receptor FAS/CD95, which belongs to the wide family of tumor necrosis factor (TNF) receptors. Although cervical cancer cells can induce the tumor-specific cytotoxic T lymphocytes (CTLs) apoptosis, the fact that an anti-CD95 antibody could block apoptosis, indicates that tumor cells induce apoptosis of CTLs through CD95-CD95 ligand (FAS/FASL) interaction [18]. Cellular apoptosis plays an important role in the human homeostasis and impaired programmed cell death is necessary for tumorigenesis [19]. Therefore, the expression of the FAS/FASL genes may represent an important element in the susceptibility for the development of cervical cancer. The most widely analysed polymorphism in cervical carcinogenesis is the FAS-670 gene promoter polymorphism (rs1800682). The rs1800682 in the promoter of the FAS gene is located within the consensus sequence of the STAT1 (signal transducer and activator of transcription 1) transcription factor binding site. The binding of the transcription factor STAT1 to the FAS gene is associated with transcriptional activation and expression of the FAS gene [20, 21]. An A to G change at FAS-670 reduces the expression of the FAS gene by abolishing the gamma interferon activation signal binding site which results in decreased activation of induced cell death [21].

The results of several studies analysing the role of the FAS-670 gene promoter polymorphism in cervical carcinogenesis are contradictory. According to the studies of Nunobiki et al. and Ueda et al. in the Japanese population, there is a higher G allele frequency in cases of high-grade squamous intra-epithelial lesions (HSIL) compared to low-grade squamous intra-epithelial lesions (LSIL) [22–24]. There is also an increased OR for GA + GG genotypes in HSIL cases compared to controls, among women infected with high-risk HPV types [22–24]. Similarly, a statistically significant correlation between the susceptibility for cervical cancer and the AG and combined AG + GG genotypes was observed in a study referring to the Indian population [25]. The frequency of the A allele and the AA genotype of the FAS-670 polymorphism was found to be higher in cases of HSIL/cervical cancer in two studies of Lai et al. [26, 27]. The same polymorphism is associated with an increased risk of cervical cancer in Brazilian women younger than 48 years old [28]. In contrast, no association between the FAS-670 polymorphism and the susceptibility to cervical cancer was shown by several studies in different populations published recently [29–31]. However, even without being correlated with susceptibility to cervical cancer, some genotypes of the FAS/FASL polymorphisms could be related to the risk of lymph node metastasis of cervical cancer [32].

The aim of our study was to evaluate the association of two SNPs of EVER1/2 genes’ region (rs2290907, rs16970849) as well as the FAS-670 polymorphism with the susceptibility to precancerous lesions and cervical cancer in a Greek population. The selection of the SNPs rs2290907and rs16970849 was based on the results of the study of Castro et al. [16] which confirmed a significant correlation between these polymorphisms and cervical cancer. The FAS-670 polymorphism was included in the present study in order to enhance the already existing and quite contradictory data concerning its association with cervical cancer.

## Methods

### Study population

In total, 525 women participated in our study. Case subjects were selected from patients treated between 2006 and 2011 at the University Clinics of Obstetrics and Gynaecology in the Aristotle University of Thessaloniki in Greece. The case group consisted of women with a medical history of precancerous lesions or cervical cancer. The control group contained healthy subjects with a history of normal cervical cytology. The recruitment of the sample and the classification between case and control subjects was based on the medical history and cytological findings of the participants, as well as their first-degree relatives. The inclusion of the participants in the case group was based on a histopathological diagnosis of precancerous lesions or cervical cancer, while the women belonging to the control group had a normal cervical cytology throughout their regular annual screening. Out of the 525 participants, 10 were excluded from the statistical analysis as none of the three polymorphisms were found in their genetic analysis. Among the 515 women who were finally included in the statistical analysis, 113 presented with precancerous lesions or cervical cancer (27 with persistent CIN1, 66 with CIN2/3 and 20 with cervical cancer) and 402 were classified to the control group. The age was not significantly different between the case and the control group. The mean age for the case group was 43.75 ± 16.33 years and the mean age for the control group was 45.2 ± 17.23 years.

All the participants were subjected to a peripheral blood test, while those that had not had a pap-test in the last year were in addition subjected to cervical cytology.

The study was approved by the local ethical review board of the Aristotle University of Thessaloniki, and all participants provided written informed consent.

### Genotyping

Genomic DNA extraction was carried out from 200 μl of EDTA-anticoagulated whole blood using a QIAamp® DNA Blood Mini Kit (Qiagen GmbH, Hilden, Germany) according to the manufacturer’s protocol. The EVER1/EVER2 (rs2290907 and rs16970849) and FAS-670 (rs1800682) SNPs were genotyped with TaqMan allele-specific PCR amplification technology on an ABI PRISM 7900 Sequence Detection System (Applied Biosystems, Foster City, USA) and analysed with the Sequence Detection Software (SDS 2.1, Applied Biosystems, Foster City, USA). Initially observed SNP allelic discrimination curves of all genotypes were confirmed by direct DNA sequencing on an ABI PRISM 3100 genetic analyser (Applied Biosystems, Foster City, USA).

Regarding the linkage-disequilibrium (LD) with other SNPs, according to the International HapMap Project, the FAS gene has three LD blocks and rs1800682 is located in the first LD block and therefore is in high LD with other SNPs within this block. Similarly, the TNRC6C gene has two LD blocks and rs2290907 is located in the second large block. Finally, TMC8 gene has four LD blocks and rs16970849 is located in the last one.

### Statistical analysis

A power analysis was performed using the CaTS Power Calculator [33]. Taking into account the number of cases and controls, our study had a power of 80% to detect odds ratios of 1.53, 1.77 and 2.55 for rs1800682, rs2290907 and rs1697084 respectively, assuming the minor allele frequencies of each SNP in the TSI population (Tuscan in Italy which is genetically closer to our population).

Basic statistical analysis for genotype-disease association was performed using the analysis tool PLINK (http://pngu.mgh.harvard.edu/purcell/plink/) [34]. The initial quality control tests included the calculation of the number of missing genotypes for each SNP and the assessment of the Hardy–Weinberg equilibrium expectation for cases and controls separately with a *p* value of 0.001 as the significance threshold. In addition, the Minor Allele Frequency (MAF) was estimated and a test looking for distortion of Mendelian segregation was performed. None of the SNPs were excluded according to the Hardy-Weinberg equilibrium test or non-Mendelian segregation. Once the quality tests were completed, a basic association analysis on an SNP by SNP base was done, comparing allele and genotype frequencies between cases and controls. The Chi-Squared test was used for detecting potential genotype-disease correlation and its associated *p*-value, as well as the odds-ratio (OR), for each SNP were calculated.

## Results

The genetic characteristics (gene, chromosome, chromosomal position, distance from the gene start, location, function and minor allele frequencies) of the three SNPs included in our study, are summarised in Table 1. The allelic as well as the genotype frequencies for all SNPs in the case and control group are summarised in Table 2. Table 3 shows the results of the chi-squared tests performed to detect any association between the three SNPs and the development of precancerous lesions or cervical cancer. The large *p* values varying between 0.26 and 0.98 indicated no statistically significant differences between the case and the control group for all the SNPs tested. The linear estimated effect sizes were 0.2, 2.2 and 55% for SNPs rs1800682, rs2290907 and rs16970849 respectively. None of them were significant.

**Table 1:** Genetic characteristics of the SNPs rs1800682, rs2290907and rs16970849

SNP	Gene	Chromosome	Chromosome Position^a^	Distance from gene start	Gene position	Function	MAF^b^
rs1800682	FAS	10	90749963	325^c^	5′ upstream	regulatory	C (0.403)
rs2290907	TNRC6C^d^	17	76093677	93483^e^	Intron 18–19	No-coding	G (0.115)
rs16970849	TMC8	17	76133908	8403^f^	Intron 11–12	No-coding	A (0.049)

^a^Chromosome positions of each SNP based on dbSNP human build 138 and human genome build 37.5

^b^MAF: minor allele frequency based on HapMap data in CEU population

^c^Distance from FAS gene start (90750288)-forward strand

^d^TNRC6C: trinucleotide repeat containing 6C gene (4 kb distance from TMC6)

^e^Distance from TNRC6Cgene start (76000194)-forward strand (15.3 kb distant from TMC6 gene start)

^f^Distance fromTMC8 gene start (76125505)-forward strand

**Table 2:** Allelic and genotype frequencies for SNPs rs1800682, rs2290907, rs16970849 in the control and case group

SNP	Genotypes/alleles	Controls, n (%)	Cases, n (%)
rs1800682			
Genotype	TT	119 (0.318)	34 (0.315)
	TC	161 (0.431)	47 (0.435)
	CC	94 (0.251)	27 (0.250)
Allele	T	399 (0.533)	115 (0.532)
	C	349 (0.467)	101 (0.468)
rs2290907			
Genotype	AA	279 (0.722)	76 (0.710)
	AG	94 (0.244)	28 (0.262)
	GG	13 (0.034)	3 (0.028)
Allele	A	652 (0.845)	180 (0.841)
	G	120 (0.155)	34 (0.159)
rs16970849			
Genotype	GG	369 (0.946)	99 (0.917)
	GA	21 (0.054)	9 (0.083)
	AA	0	0
Allele	G	759 (0.973)	207 (0.958)
	A	21 (0.027)	9 (0.042)

**Table 3:** Allelic-based analysis in the control and case group for the SNPs rs1800682, rs2290907, rs16970849

SNP	Case group frequency of minor allele	Control group frequency of minor allele	Chi-square test	*P* value	OR (95% CI)
rs1800682	0.4676	0.4666	0.0006938	0.979	1.004 (0.741–1.360)
rs2290907	0.1589	0.1554	0.01503	0.902	1.026 (0.678–1.554)
rs16970849	0.04167	0.02692	1.259	0.262	1.571 (0.709–3.483)

The lack of association between the studied SNPs and cervical cancer was further confirmed by the large *p* values obtained by the genotype-based analysis (Table 4) as well as the dominant and recessive model analysis (Table 5). Both comparisons failed to detect any statistically significant difference between the case and the control group. At this point it should be mentioned that no results were obtained for the recessive model in the case of the SNP rs16970849, due to the fact that there were no women with AA genotype detected in the study sample.

**Table 4:** Genotype-based analysis in the control and case group for the SNPs rs1800682, rs2290907, rs16970849

SNP	Genotype	Controls	Cases	*p*-value	OR (95% CI)
rs1800682	aa(CC)	94(25.1%)	27(25.0%)	0.996	0.955(0.539–1.690)
	Aa(TC)	161(43.1%)	47(43.5%)		1.022(0.619–1.686)
	AA (TT)	119(31.8%)	34(31.5%)		1
rs2290907	aa(GG)	13(3.4%)	3(2.8%)	0.8994	0.847(0.235–3.049)
	Aa(AG)	94(24.3%)	28(26.2%)		1.094(0.668–1.789)
	AA(AA)	279(72.3%)	76(71.0%)		1
rs16970849	aa(AA)	0(0%)	0(0%)	0.2544	-
	Aa(GA)	21(5.4%)	9(8.3%)		1.597(0.709–3.597)
	AA(GG)	369(94.6%)	99(91.7%)		1

**Table 5:** Dominant/recessive model in the control and case group for the SNPs rs1800682, rs2290907, rs16970849

SNP	Genotype	Controls	Cases	Dominant model (aa/Aa versus AA) *p*-value	Recessive model (aa versus AA/Aa) *p*-value
rs1800682	aa(CC)	94(25.1%)	27(25.0%)	0.9472	0.9775
	Aa(TC)	161(43.1%)	47(43.5%)		
	AA (TT)	119(31.8%)	34(31.5%)		
rs2290907	aa(GG)	13(3.4%)	3(2.8%)	0.7986	0.7708
	Aa(AG)	94(24.3%)	28(26.2%)		
	AA(AA)	279(72.3%)	76(71.0%)		
rs16970849	aa(AA)	0(0%)	0(0%)	0.2544	–
	Aa(GA)	21(5.4%)	9(8.3%)		
	AA(GG)	369(94.6%)	99(91.7%)		

A supplementary analysis was performed comparing the control group with each one of the following subgroups: CIN1/CIN2, CIN2/CIN3 and CIN3/Cervical cancer. Borderline significant difference (*p* value = 0.079) was only found by the allelic model between the control group and the CIN1/CIN2 patients’ subgroup for the polymorphism rs16970849. No statistically significant difference was detected by the comparison of the other subgroups with the control group.

According to the results of the statistical analysis none of the SNPs included in our study can be associated with statistical significance with the development of precancerous lesions or cervical cancer.

## Discussion

The aim of our study was to evaluate the potential contribution of certain EVER1/2 and FAS polymorphisms to the susceptibility for cervical cancer. None of the three SNPs investigated in the present study (rs1800682, rs2290907 and rs16970849) present any statistically significant association with the development of persistent precancerous lesions or cervical cancer.

Current literature data regarding EVER genes presume their participation in the control of the HPV life cycle and pathogenesis as well as their influence on the host’s immune responses and, therefore, its capacity for HPV clearance [35, 36]. According to the model proposed by Lazarczyck et al., the interaction between the E5 protein of the HPV with the TMC/ZnT complex in keratinocytes could lead to an increased level of intracellular free Zn^2+^ with a consequent increased activity of transcription factors and viral replication [37]. The regions of EVER1/TMC6 and EVER2/TMC8 have been previously reported as interesting candidate genes in persistent viral infections and cervical cancer [14]. The SNP-based analysis of Wang et al. revealed a statistically significant association of the SNP rs9893818 with cervical precancer and cancer, but not with HPV persistence [14].

The selection of the SNPs rs2290907and rs16970849 was based on the association of these polymorphisms with susceptibility to cervical cancer, shown recently by Castro et al. [16]. This was the largest study to date to analyse the role of EVER polymorphisms in the development of cervical cancer by examining the 58 kb genomic region harbouring the TMC6 and TMC8 genes in the Swedish population. The sample size of this study was 2989 cases and 2281 controls, with 93% of the cases diagnosed with CIN3 and 7% with ICC. Among the 22 genotyped SNPs, rs2290907 and rs16970849 were found to be significantly associated with cervical cancer (rs2290907: OR GGvsAA = 0.6, 95% CI: 0.3–0.9, *p* = 0.02 and rs16970849: OR AGvsGG = 0.8, 95% CI: 0.66–0.98, *p* = 0.03). In contrast to the study of Castro et al., our study did not detect any statistically significant association between these polymorphisms and susceptibility to cervical cancer. The ethnically distinct populations could be a possible explanation to the opposed results of the two studies. In fact, the genotype frequencies of the SNP rs16970849 in our sample were in the opposite direction from that noted in the study of the Swedish population. GG genotype was overrepresented in the control group in our study (94.6%) and in the case group in the study of Castro et al. (90.2%). This might reflect the differences in the genetic background between the two populations. Since this is the first study investigating the association of the EVER genes with cervical cancer in a Greek population, no other data from an ethnically similar sample are available for comparison.

The FAS gene, belonging to the TNF receptors superfamily, has been related to an altered apoptosis procedure and potential failure to induce apoptosis of HPV-infected cells. The association between the cell surface death receptor FAS and cervical cancer has been widely studied in the past with most of the studies resulting in conflicting findings. Two recent meta-analyses, performed to clarify the already existing contradictory data, show no association between the most investigated FAS-670 polymorphism and susceptibility to cervical cancer in Caucasian, Asian and African populations [38, 39]. Shen et al. included ten studies in the meta-analysis with a total of 4,904 participants (2,317 cases and 2,587 controls). After calculating the ORs for the allele, the homozygous, the dominant and the recessive genetic models, they concluded that there is no association between FAS-670 polymorphism and susceptibility to cervical cancer in both Caucasian and Asian women [38]. Similarly, the meta-analysis performed by Chen et al., consisting of seven studies (1856 cervical cancer patients and 2097 controls), showed a lack of association of the FAS-670 polymorphism with cervical cancer [39]. The findings of our study agree with the conclusions of these two recently published meta-analyses, validating the absence of association between the FAS-670 promoter polymorphism and susceptibility to persistent precancerous lesions and development of cervical cancer in the Greek female population.

To our knowledge this is the first study in the Greek population. Since it was designed as a hospital-based study and was performed in a relatively small sample, the conclusions may be susceptible to selection bias. Furthermore, the inclusion of women with precancerous lesions in the group of cases may not have been as strong as a group of patients exclusively with cervical cancer. Nevertheless, the aim of our study was to detect any possible association of certain polymorphisms with persistent HPV infection, which could be seen in cases of precancerous lesions as well as cases of cervical cancer. Finally, another type of limitation to consider is the small sample size of our study, which could not allow the declaration of a null result from the present study.

## Conclusions

The present study did not show any significant association of the EVER1/2 polymorphisms (rs2290907and rs16970849) with cervical cancer. However, it does provide additional data favouring no association between the FAS polymorphism (rs1800682) and the susceptibility to persistent precancerous lesions and cervical cancer. Current literature data for EVER1/2 polymorphisms and cervical cancer are very limited worldwide, therefore larger scale prospective studies are necessary in order to further clarify the role of these polymorphisms in cervical carcinogenesis.

## Abbreviations

CIN1/2/3: Cervical intraepithelial neoplasia grade 1/2/3
CTLs: Cytotoxic T lymphocytes
EV: Epidermodysplasia verruciformis
HPV: Human papillomavirus
HSIL/LSIL: High/low grade squamous intra-epithelial lesions
ICC: Invasive cervical carcinoma
LD: Linkage-disequilibrium
MAF: Minor allele frequency
OR: Odds-ratio
SNPs: Single nucleotide polymorphisms
STAT1: Signal transducer and activator of transcription 1
TMC6/8 gene: Transmembrane channel-like protein 6/8 gene
TNF: Tumor necrosis factor
